# Epidemiology of pneumonia in hospitalized adults ≥18 years old in four districts of Ulaanbaatar, Mongolia, 2015–2019

**DOI:** 10.1016/j.lanwpc.2022.100591

**Published:** 2022-09-10

**Authors:** Kirsten Fagerli, Mukhchuluun Ulziibayar, Bujinlkham Suuri, Dashtseren Luvsantseren, Dorj Narangerel, Purevsuren Batsaikhan, Bilegtsaikhan Tsolmon, Bradford D. Gessner, Eileen M. Dunne, Anneke C. Grobler, Cattram D. Nguyen, Tuya Mungun, E. Kim Mulholland, Claire von Mollendorf

**Affiliations:** a University of Melbourne, Melbourne, Australia; b Murdoch Children's Research Institute, Melbourne, Australia; c The National Centre for Communicable Disease, Ministry of Health, Bayanzurkh distrct, Horoo 14, 13th district, Nam Yan Ju Street, Ulaanbaatar 210648, Mongolia; d Ministry of Health, WW8C+79C, Olympic Street, Ulaanbaatar, Mongolia; e Pfizer Inc., 500 Arcola Rd., Collegeville, PA 19426, USA; f London School of Hygiene and Tropical Medicine, London, UK

**Keywords:** Pneumonia, Hospitalization, Adult pneumonia, Disease incidence, Mongolia

## Abstract

**Background:**

Community-acquired pneumonia is a leading cause of morbidity and mortality among children and adults worldwide. Adult pneumonia surveillance remains limited in many low- and middle-income settings, resulting in the disease burden being largely unknown.

**Methods:**

A retrospective cohort study was conducted by reviewing medical charts for respiratory admissions at four district hospitals in Ulaanbaatar during January 2015–February 2019. Characteristics of community-acquired pneumonia cases were summarized by disease severity and age. To explore factors associated with severe pneumonia, we ran univariable and age-adjusted logistic regression models. Incidence rates were calculated using population denominators.

**Results:**

In total, 4290 respiratory admissions met the case definition for clinical pneumonia, including 430 admissions of severe pneumonia. The highest proportion of severe pneumonia admissions occurred in adults >65 years (37.4%). After adjusting for age, there were increased odds of severe pneumonia in males (adjusted odds ratio [aOR]: 1.63; 95% confidence interval [CI]: 1.33-2.00) and those with ≥1 underlying medical condition (aOR: 1.46; 95% CI: 1.14-1.87). The incidence of hospitalized pneumonia in adults ≥18 years increased from 13.49 (95% CI: 12.58-14.44) in 2015 to 17.65 (95% CI: 16.63-18.71) in 2018 per 10,000 population. The incidence of severe pneumonia was highest in adults >65 years, ranging from 9.29 (95% CI: 6.17-13.43) in 2015 to 12.69 (95% CI: 9.22-17.04) in 2018 per 10,000 population.

**Interpretations:**

Vaccination and other strategies to reduce the risk of pneumonia, particularly among older adults and those with underlying medical conditions, should be prioritized.

**Funding:**

Pfizer clinical research collaboration agreement (contract number: WI236621).

**Research in context**

**Evidence before this study**

Community-acquired pneumonia is a leading cause of morbidity and mortality among both children and older adults worldwide. In many high-income countries, the burden of community-acquired pneumonia is well researched, with many countries having robust surveillance systems in place. However, respiratory disease surveillance remains limited in low- and middle-income countries (LMICs). Additionally, a majority of published literature on pneumococcal and other respiratory diseases tends to focus on the childhood burden of disease, especially in Asia. We conducted a PubMed search between 2-6 August 2021 and again on 7 July 2022 for studies describing adult community-acquired pneumonia in Asian or other LMICs with the following search terms ((pneumonia) AND (adult OR >65 OR ≥18 OR elderly) AND (Mongolia OR Asia OR LMIC) AND (burden OR determinant OR surveillance)). Studies that focused on disease characteristics related to the SARS-Cov-2 virus and the COVID-19 pandemic were excluded, as our study period occurred prior to the pandemic. A majority of studies were disease-specific with pneumonia being one potential outcome. Six studies in Asia examined the influenza epidemiology or burden of disease, including one study in Mongolia. Two studies focused on atypical pathogens associated with pneumonia. An additional six studies focused on antimicrobial resistance or on serotype replacement post-introduction of pneumococcal vaccination in children. Only three studies from other Asian LMICs focused on the burden of hospitalized pneumonia in adults. No studies systematically studied the burden or determinants of adult pneumonia in Mongolia.

**Added value of this study**

To our knowledge, this is the first systematic surveillance program examining pneumonia in adults in Mongolia. Notably, we found the incidence for clinical and severe clinical pneumonia hospitalizations to be at least three times higher in adults >65 years than in other age groups. Adults with underlying medical conditions were more likely to have severe clinical pneumonia compared to patients without underlying conditions. Our findings also further describe the indicators of pneumonia severity in the capital city of Ulaanbaatar.

**Implications of all the available evidence**

This study presents important epidemiological information about a previously unknown pneumonia burden in an adult population in Asia. As the proportion of the elderly population in Mongolia is expected to rapidly grow in the coming years, it is likely that pressures on the country's healthcare system will also increase. Our findings suggest that older adults and those with underlying medical conditions are most at risk for developing pneumonia, and with higher disease severity, than other groups. By establishing the adult burden and determinants of pneumonia, targeted interventions can be developed to have a more meaningful impact on the burden of disease.

Alt-text: Unlabelled box

## Introduction

Community-acquired pneumonia is a leading cause of morbidity and mortality worldwide.^1^^,^^2^ In adult populations, pneumonia disproportionally affects individuals >65 years and those with underlying medical conditions, who often endure high rates of hospitalizations and mortality.^3^^,^^4^ Smoking status, alcohol intake, comorbidities, and air pollution exposure are important risk factors that contribute to the pneumonia burden.^5^^,^^6^ Immunosenescence may also render older adults more susceptible to colonization and infection by pneumonia-causing organisms.^3^

Many respiratory pathogens are known to cause community-acquired pneumonia including *Streptococcus pneumoniae*, influenza, and respiratory syncytial virus. In adults, *S. pneumoniae* is the leading cause of community-acquired pneumonia, with one meta-analysis attributing 27% of pneumonia cases to the pneumococcus.^2^^,^^7^^,^^8^

While the burden of community-acquired pneumonia is well understood in many high-income countries, respiratory disease surveillance remains limited in low- and middle-income countries (LMICs).^2^^,^^7^^,^^9^^,^^10^ Furthermore, published data on pneumococcal and other respiratory diseases tend to focus on the childhood burden.^4^ In the Asia-Pacific region, a gap in understanding the adult burden of community-acquired pneumonia persists, with a recent review finding that the majority of available data were primarily from higher-income countries and those with well-established vaccination programs.^4^

Mongolia is a lower-middle income country in central Asia, with an estimated population of 3·3 million people.^11^ In 2018, the average life expectancy in Mongolia was 71·7 years, with almost two-thirds of its population aged between 15–64 years and 4·1% of the population >65 years.^11^^,^^12^ Respiratory diseases are a leading cause of morbidity in Mongolia, with pneumonia comprising 51·8% of respiratory admissions across all ages in 2018.^11^ High levels of air pollution, high prevalence of cigarette smoking, and cold winter temperatures contribute to Mongolia's pneumonia burden.^13^, ^14^, ^15^ Currently, there are no recommendations for adult pneumococcal vaccination in Mongolia and a limited number of influenza vaccines are available to high-risk individuals.^16^

To address the high burden of pediatric pneumonia, the Mongolian government introduced the 13-valent pneumococcal conjugate vaccine (PCV13) into the childhood vaccination schedule from June 2016.^17^ An expanded pneumonia surveillance program was established in the capital city of Ulaanbaatar (∼1·5 million population) to better understand the burden of all-cause pneumonia in children 2–59 months old, finding high rates of pneumonia.^18^ A subsequent adult surveillance program was established in 2019, as part of a larger study aiming to determine the indirect effect of childhood PCV13 on community-acquired pneumonia in adults.^19^ To quantify the burden of adult community-acquired pneumonia in Ulaanbaatar, hospital-based surveillance was initiated in four districts of the city. Our study aims to establish the burden and clinical characteristics of community-acquired pneumonia in adults 18 years or older in Mongolia.

## Methods

### Study design

Data were collected as part of an ongoing hospital-based community-acquired pneumonia surveillance program for adults ≥18 years in four districts of Ulaanbaatar: Songinokhairkhan, Sukhbaatar, Bayanzurkh, and Chingeltei. These are the largest districts in Ulaanbaatar, accounting for approximately 70% of the city's population, with a mixture of formal and traditional (colloquially known as ger) housing.^17^

Surveillance data were collected both retrospectively (January 2015–February 2019) and prospectively (March 2019–March 2022).^19^ For the purposes of this analysis, only retrospective data were included.

### Data collection

We constructed a retrospective cohort by conducting medical chart reviews for adult community-acquired pneumonia admissions. Trained staff reviewed hospital admission logs to identify all respiratory admissions. All medical records for respiratory admissions were reviewed to confirm whether the patient had community-acquired pneumonia. Details on eligibility and reasons for exclusion were captured on a screening log. All intensive care unit (ICU) admissions and deaths were also reviewed to ensure that severe disease cases were counted.

Clinical pneumonia was defined as a patient who was diagnosed with community-acquired pneumonia at both admission and at time of discharge, and with two or more of the following symptoms (at least one of which must be respiratory):^7^^,^^19^

• reported fever or measured fever (≥38·0°C)
• new cough or change in chronic cough
• new or increased sputum production
• leucocytosis (>11·0 × 10^9^ white blood cells/l) or leukopenia (<4·0 × 10^9^ white blood cells/l)
• dyspnoea or tachypnoea (i.e., difficult breathing or respiratory rate >25 breaths/minute)
• lung findings on auscultation
• hypoxaemia (oxygen saturation <90%)
• pleuritic chest pain

Severe clinical pneumonia was defined as clinical pneumonia with 1) an ICU admission, 2) death during hospital stay, or 3) clinical pneumonia with two or more of the following symptoms:^19^^,^^20^

• impaired conscious state (Glasgow coma scale <15)
• respiratory rate of ≥30 breaths per minute
• hypotension (systolic blood pressure ≤90 mmHg or diastolic blood pressure ≤60 mmHg)
• hypoxaemia

Patients that met the case definition criteria were included in the study. Staff extracted relevant clinical data from patient medical charts, including clinical presentation at admission and case management, age, underlying medical conditions, and clinical outcomes. Documented underlying medical conditions included asthma, chronic obstructive pulmonary disease (COPD)/emphysema, tuberculosis, cirrhosis/liver failure, coronary artery disease, hypertension, heart failure, chronic renal failure, and diabetes. Study case report forms reported a patient as having sequelae if the patient still had any pneumonia-related symptoms or any subsequent complications related to their hospitalization at the time of discharge, based on the clinical judgement of treating physicians. If a patient meeting the study inclusion criteria was readmitted to the hospital for community-acquired pneumonia within 14 days of their previous admission, the subsequent record was considered to be part of the same pneumonia episode and excluded from analysis.

### Statistical analysis

Data were analyzed using Stata version 16·0 (StataCorp LP, College Station, TX). Case characteristics were classified by disease severity and age, and were summarized by either percentages or medians with interquartile ranges (IQR). For variables with missing data, summary statistics were calculated among those with available data. For all variables other than documented underlying medical conditions and reported antibiotic use, <5% of data were missing from analysis. Data were >85% complete for reported antibiotic use and between 46%–59% complete for individual comorbidities. To explore factors associated with severe pneumonia among admitted patients, we first ran univariable logistic regressions, where each model treated severe pneumonia as the outcome variable and each candidate factor as an independent variable. Because the association between community-acquired pneumonia and older age has been previously reported, we adjusted for age groups (18–25, 26–45, 46–65, >65 years) in additional models to account for potential confounding.^3^

We calculated cumulative incidence rates with Poisson confidence intervals, stratified by age group, for all clinical pneumonia admissions using population denominators for the four districts provided by the Mongolian Health Department census for each year from 2015 to 2018 inclusive. Incidence rates for 2019 were not calculated because the retrospective surveillance concluded in February 2019. To examine the potential impacts of incomplete data on the disease burden, a post-hoc sensitivity analysis was completed. This analysis explored the upper limit of what the incidence would be if all patients with a community-acquired pneumonia diagnosis at admission, but inadequate medical records for study inclusion, had met our case definition for clinical pneumonia.

### Ethical review

The protocol was approved by the Human Research Ethics committees of the Mongolian National Ethics Committee for Health Research and the Royal Children's Hospital, Melbourne (HREC 38045).

## Results

**Figure 1:**
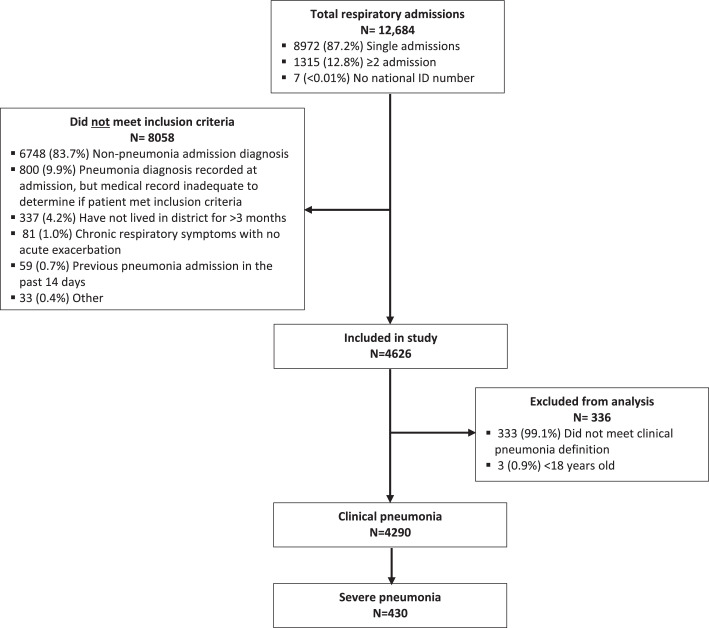
Inclusion of study participants with respiratory-related admissions at four sentinel hospitals in Ulaanbaatar, Mongolia, January 2015– February 2019.

**Figure 2:**
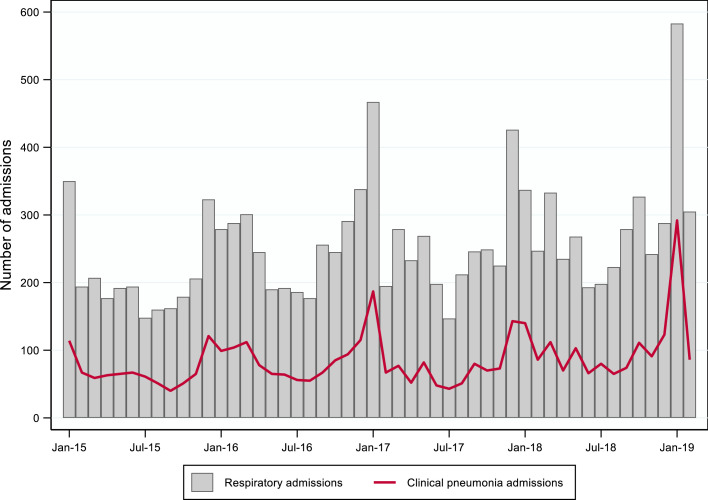
Total respiratory and clinical pneumonia hospital admissions in adults ≥18 years old, reported through retrospective chart review, Ulaanbaatar, Mongolia, January 2015–February 2019.

In total, 12,684 respiratory admissions were screened at four district hospitals (Figure 1). Of these, 8058 admissions did not meet the inclusion criteria, including 800 admissions (9·9%) where a community-acquired pneumonia diagnosis had been recorded at admission, but medical records were inadequate to determine if patients met inclusion criteria. A further 336 admissions were excluded from analysis because they had not met the case definition. Of 4290 admissions that met the case definition for clinical pneumonia, 430 (10·0%) admissions were classified as severe clinical pneumonia. There were 3,832 (94·7%) patients included once in the study, 191 (4·7%) twice, 24 (0·6%) three times, and one (0·02%) included four times. Of 3459 patients with influenza immunization histories reported on their medical charts, 11 (0·3%) had received influenza vaccinations within the past 12 months. Of 4121 patients with pneumococcal immunization histories reported, three (0·07%) reported receiving either the pneumococcal polysaccharide vaccine or PCV13 within 30 days of admission, but this could not be verified. Clinical pneumonia hospitalizations showed a seasonal pattern, with the highest number of cases presenting over Mongolia's winter months (November–March) and lowest number of cases presenting during the summer months (June–August) (Figure 2). Most adults >65 years (84·2%) had a documented underlying medical condition; underlying medical conditions were reported less frequently in younger age groups: 18-25 years (6·7%), 26–45 years (20·1%), and 46–65 years (61·5%).

### Factors associated with disease severity

While adults 26-45 years made up the highest percentage (34·3%) of all clinical pneumonia admissions, adults >65 years made up most cases of severe pneumonia (37·4%) (Table 1). Females accounted for more than half of all clinical pneumonia admissions (58·4%); however, just over half of severe pneumonia cases were men (52·6%). Approximately one-third (34·3%) of all clinical pneumonia cases and a quarter (23·6%) of severe pneumonia cases had taken an antibiotic within 48 hours of hospital presentation. Almost two-thirds (64·4%) of severe pneumonia admissions had ≥1 underlying medical condition.

**Table 1:** Characteristics of adults hospitalized with community-acquired pneumonia included in retrospective medical chart review, by pneumonia severity, Ulaanbaatar, Mongolia, January 2015– February 2019.

	All pneumonia admissions *N* = 4290	Clinical (non-severe) pneumonia *N* = 3860	Severe clinical pneumonia *N* = 430
Age (years), median (interquartile range [IQR])	49 (32–63)	47 (32–62)	58 (44–74)
Age groups (years), n (%)			
18–25	465 (10.8)	451 (11.7)	14 (3.3)
26–45	1471 (34.3)	1370 (35.5)	99 (23.0)
46–65	1423 (33.2)	1267 (32.8)	156 (36.3)
>65	931 (21.7)	770 (20.0)	161 (37.4)
Sex, n (%)			
Female	2506 (58.4)	2302 (59.6)	204 (47.4)
Male	1784 (41.6)	1558 (40.4)	226 (52.6)
Hospital surveillance district, n (%)			
Bayanzurkh	1250 (29.1)	1096 (28.4)	154 (35.8)
Songinokhairkhan	1537 (35.8)	1440 (37.3)	97 (22.6)
Sukhbaatar	1044 (24.3)	948 (24.6)	96 (22.3)
Chingeltei	459 (10.7)	376 (9.7)	83 (19.3)
Reported antibiotic use within 48hrs of hospital presentation, n (%)	1263/3682 (34.3)	1184/3347 (35.4)	79/335 (23.6)
Underlying medical conditions, n (%)			
≥1 underlying medical condition^a^	1985 (46.3)	1708 (44.3)	277 (64.4)
Asthma	120/2105 (5.7)	109/1863 (5.9)	11/242 (4.6)
COPD^b^/emphysema	143/1989 (7.2)	101/1759 (5.7)	42/230 (18.3)
Tuberculosis	139/2501 (5.6)	113/2209 (5.1)	26/292 (8.9)
Cirrhosis/liver failure	121/2434 (5.0)	99/2176 (4.6)	22/258 (8.5)
Coronary artery disease	548/2006 (27.3)	442/1768 (25.0)	106/238 (44.5)
Hypertension	1334/2342 (57.0)	1185/2085 (56.8)	149/257 (58.0)
Heart failure	182/2445 (7.4)	134/2166 (6.2)	48/279 (17.2)
Chronic renal failure	181/2518 (7.2)	140/2230 (6.3)	41/288 (14.2)
Diabetes	316/2341 (13.5)	253/2073 (12.2)	63/268 (23.5)

a Based on documentation in medical chart.

b Chronic obstructive pulmonary disease.

On univariable analysis, age, sex, and underlying medical conditions were associated with disease severity (Table 2). Patients >65 years had an almost seven-fold increase in the odds of severe clinical pneumonia compared with patients 18–25 years of age. After adjusting for age, males had increased odds of severe pneumonia compared with females (adjusted odds ratio [aOR]: 1·63; 95% CI: 1·33–2·00). In addition, several underlying medical conditions were associated with increased odds of severe pneumonia, and patients that had ≥1 documented underlying medical condition had higher odds of severe pneumonia than adults without any underlying medical conditions (aOR: 1·46; 95% CI: 1·14-1·87) (Table 2). Patients with hypertension had decreased odds of severe pneumonia (aOR: 0·55; 95% CI: 0·40-0·76). Patients who had received antibiotics within 48 hours prior to hospital admission had lower odds of severe pneumonia than those who had not taken antibiotics (aOR: 0·60; 95% CI: 0·46-0·78).

**Table 2:** Univariable and age-adjusted analysis of factors associated with severity of illness among patients with community-acquired pneumonia included through retrospective medical chart review, Ulaanbaatar, Mongolia, January 2015–February 2019.

	Odds ratio (95%CI)	Age-adjusted odds ratio (95% CI)
Age (years)		
18–25	Ref.	NA
26–45	2.32 (1.32-4.11)	NA
46–65	3.97 (2.27-6.93)	NA
>65	6.74 (3.85-11.77)	NA
Sex		
Female	Ref.	Ref.
Male	1.64 (1.34-2.00)	1.63 (1.33-2.00)
Antibiotics given within 48hrs prior to hospital admission	0.56 (0.43-0.73)	0.60 (0.46-0.78)
Underlying medical conditions		
≥1 underlying medical condition^a^	2.28 (1.85-2.81)	1.46 (1.14-1.87)
Asthma	0.77 (0.41-1.45)	0.74 (0.39-1.39)
COPD^b^/emphysema	3.67 (2.48-5.42)	2.72 (1.81-4.08)
Tuberculosis	1.81 (1.16-2.83)	2.11 (1.34-3.32)
Cirrhosis/liver failure	1.96 (1.21-3.16)	1.85 (1.14-3.01)
Coronary artery disease	2.41 (1.83-3.18)	1.41 (1.00-1.99)
Hypertension	1.05 (0.81-1.36)	0.55 (0.40-0.76)
Heart failure	3.15 (2.21-4.50)	2.32 (1.60-3.35)
Chronic renal failure	2.48 (1.71-3.60)	2.17 (1.49-3.17)
Diabetes	2.21 (1.62-3.02)	1.97 (1.43-2.71)

CI = Confidence Interval.

a Based on documentation in medical chart.

b Chronic obstructive pulmonary disease.

### Indicators of disease severity

Of 315 patients admitted to the ICU, 296 (94·0%) required oxygen therapy, 9 (2·9%) had a chest drain inserted, 78 (24·8%) required vasopressor therapy, and 313 (99·4%) were given antibiotics. Forty-five of 46 (97·8%) patients who died had been admitted to the ICU.

Patients >65 years had the highest percentage of ICU admissions (13·6%) and remained in the ICU for a longer duration (median: 3 days; IQR: 2-4 days) than other age groups (Table 3). Young adults (18-25 years old) were admitted to the ICU least frequently (1·1%). Adults >65 years accounted for the highest proportion of patients that required oxygen therapy (21·4%) and vasopressor therapy (3·5%) and stayed in the hospital longer duration (median: 8 days; IQR: 7 – 9 days) than other age groups.

**Table 3:** Clinical characteristics of adults hospitalized with community-acquired pneumonia included in retrospective medical chart review, by age group, Ulaanbaatar, Mongolia, January 2015– February 2019.

	All pneumonia admissions *N* = 4290	18–25 years *N =* 465	26–45 years *N =* 1471	46–65 years *N =* 1423	>65 years *N* = 931
Severe clinical pneumonia, n (%)	430 (10.0)	14 (3.0)	99 (6.7)	156 (11.0)	161 (17.3)
Patient admitted to ICU^a^, n (%)	315 (7.3)	5 (1.1)	68 (4.6)	115 (8.1)	127 (13.6)
Duration of ICU^a^ stay (days), median (IQR)	2 (1–4)	2.5 (1.5–3)	2 (1-4)	2 (1–3)	3 (2-4)
Oxygen therapy provided, n (%)	505/4284 (11.8)	11 (2.4)	96/1469 (6.5)	199/1421 (14.0)	199/929 (21.4)
Duration of oxygen therapy (days), median (IQR)	2 (1–3)	1 (1–2)	2 (1–3)	2 (1–2)	2 (1–3)
Chest drain inserted, n (%)	18/4289 (0.4)	1/464 (0.2)	8 (0.5)	4 (0.3)	5 (0.5)
Duration of chest drain (days), median (IQR)	3 (2–3)	2 (2–2)	3 (2–4)	1.5 (1–2)	3 (3–4)
Vasopressor therapy given, n (%)	101 (2.4)	1 (0.2)	22 (1.5)	45 (3.2)	33 (3.5)
Duration of vasopressor therapy (days), median (IQR)	2 (1–3)	1 (1–1)	1 (1–2)	3 (1–3)	3 (1–4.5)
Antibiotics given in hospital, n (%)	4283/4288 (99.9)	465 (100)	1468/1470 (99.9)	1420/1422 (99.9)	930 (99.9)
Duration of antibiotic therapy (days), median (IQR)	5 (5–6)	5 (5–6)	5 (5–6)	5 (5–6)	5 (5–6)
Length of hospital stay (days), median (IQR)	7 (7–9)	7 (7–9)	7 (7–9)	7 (7–9)	8 (7–9)
**Disease outcome, n (%)**	*N=*4279	*N=*464	*N=*1470	*N=*1419	*N=*926
Recovered without sequelae	1847 (43.2)	285 (61.4)	834 (56.7)	566 (39.9)	162 (17.5)
Recovered with sequelae^b^	2311 (54.0)	173 (37.3)	600 (40.8)	813 (57.3)	725 (78.3)
Death	46 (1.1)	0 (0)	11 (0.8)	19 (1.3)	16 (1.7)
Transferred to another hospital^c^	75 (1.8)	6 (1.3)	25 (1.7)	21 (1.5)	23 (2.5)

IQR = Interquartile range.

a Intensive care unit.

b Defined as any pneumonia-related symptoms or any subsequent complications related to their hospitalization at the time of discharge, based on the clinical judgement of treating physicians.

c Health outcome after transfer not available.

### Case-fatality ratio

We obtained health outcome data for 4,279 of 4,290 clinical pneumonia hospitalizations (Table 3). Of these, 4,158 (97·2%) recovered with or without sequelae and 75 (1·8%) were transferred to another hospital; health outcome data were unavailable after transfer. There were 46 documented deaths during our study period (case-fatality rate: 1·1%; 95% CI: 0·8 – 1·4%). Eleven (0·8%) patients 26–45 years old died, 19 (1·3%) patients 46–65 years old died, and 16 (1·7%) patients >65 years old died. No deaths were reported in the 18–25 years age group.

Among deaths in the 26-45 years age group, just over half (54·6%) had ≥1 documented underlying medical condition. The majority of patients aged 46–65 years and >65 years who died also had ≥1 underlying medical condition (73·7% and 87·5%, respectively). All but one patient had been admitted to the ICU, with a median length of ICU stay of 2 days (IQR: 1 – 5·5 days).

More than half (54·0%) of all patients were discharged with sequelae, with two-thirds of these patients being >45 years (66·6%). Having sequelae at time of discharge was most frequently reported in patients >65 years (78·3%); young adults reported having sequelae least frequently (37·3%).

### Pneumonia incidence rates

**Figure 3:**
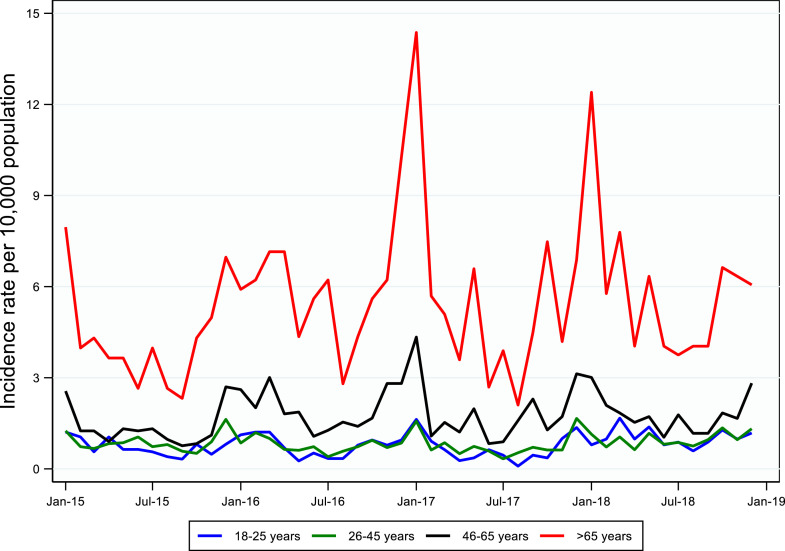
Monthly incidence rate of community-acquired pneumonia hospital admissions in adults ≥18 years old, by age group, reported through retrospective chart review, Ulaanbaatar, Mongolia, January 2015–December 2018.

Adults >65 years had the highest monthly incidence for clinical pneumonia hospitalizations, followed by adults 46–65 years (Figure 3). Adults 18–25 years consistently reported the lowest monthly incidence rates for the duration of the study period. Similarly, the annual incidence for severe pneumonia hospitalizations was highest in older age groups and declined with age (Table 4). For adults >65 years, the incidence for all clinical pneumonia hospitalizations increased from 51·42 (95% CI: 43·65 – 60·18) per 10,000 population in 2015 to 71·24 (95% CI: 62·63–80·70) per 10,000 population in 2018, and the incidence for severe pneumonia hospitalizations increased from 9·29 (95% CI: 6·17 – 13·43) per 10,000 population in 2015 to 12·69 (95% CI: 9·22 – 17·04) per 10,000 population in 2018. For young adults, the incidence was comparably lower for all clinical pneumonia hospitalizations (8·54 [95% CI: 6·99 – 10·33] in 2015 and 12·39 [95% CI: 10·32 – 14·75] in 2018, per 10,000 population) and for severe pneumonia hospitalizations (0·32 [95% CI: 0·09 - 0·82] in 2015 and 0·10 [95% CI: 0 - 0·55] in 2018, per 10,000 population).

**Table 4:** Incidence rates for community-acquired pneumonia hospitalizations by age and disease severity in adult ≥18 years of age in Ulaanbaatar, Mongolia, 2015–2018 (per 10,000 population).

	All clinical pneumonia				Severe clinical pneumonia			
	2015IR (95% CI)	2016IR (95% CI)	2017IR (95% CI)	2018IR (95% CI)	2015IR (95% CI)	2016IR (95% CI)	2017IR (95% CI)	2018IR (95% CI)
18–25 years	8.54 (6.99-10.33)	9.13 (7.47-11.04)	8.16 (6.56-10.03)	12.39 (10.32-14.75)	0.32 (0.09-0.82)	0.34 (0.09-0.88)	0.27 (0.06-0.80)	0.10 (0-0.55)
26–45 years	10.54 (9.44-11.74)	9.21 (8.21-10.31)	9.35 (8.34-10.44)	11.71 (10.58-12.93)	0.83 (0.54-1.22)	0.70 (0.44-1.05)	0.59 (0.36-0.91)	0.54 (0.32-0.85)
46–65 years	16.20 (14.19-18.41)	23.87 (21.46-26.48)	21.88 (19.63-24.33)	21.67 (19.47-24.06)	1.94 (1.29-2.80)	2.41 (1.69-3.33)	3.00 (2.20-3.99)	2.09 (1.45-2.92)
>65 years	51.42 (43.65-60.18)	71.86 (62.89-81.75)	67.05 (58.55-76.43)	71.24 (62.63-80.70)	9.29 (6.17-13.43)	13.06 (9.42-17.66)	10.78 (7.55-14.92)	12.69 (9.22-17.04)
*All ≥18 years*	*13.49 (12.58-14.44)*	*15.91 (14.94-16.93)*	*15.24 (14.30-16.23)*	*17.65 (16.63-18.71)*	*1.41 (1.12-1.74)*	*1.68 (1.37-2.03)*	*1.66 (1.36-2.01)*	*1.53 (1.24-1.87)*

IR= Incidence rate; CI = Confidence Interval.

The overall incidence for all clinical pneumonia hospitalizations in adults ≥18 years increased from 13·49 (95% CI: 12·58 - 14·44) in 2015 to 17·65 (95% CI: 16·63 - 18·71) in 2018, per 10,000 population (Table 4). In a sensitivity analysis that included all pneumonia admissions whose medical records were inadequate for study inclusion from 2015–2018 (*n=*751), we found that the incidence of pneumonia may be up to ∼20% higher than our more conservative estimates (S1 Table).

## Discussion

This study describes the burden and epidemiology of community-acquired pneumonia in adults ≥18 years in four districts of Ulaanbaatar, Mongolia over a four-year period. While adults 26-45 years made up the highest percentage of pneumonia admissions, severe pneumonia was most common among adults >65 years. Male gender and underlying medical conditions were also associated with severe pneumonia.

In 2018, the incidence of adults hospitalized with clinical pneumonia was 17·65 per 10,000 population, lower than that of several high-income countries, including the USA (24·8 per 10,000 population), Germany (97 per 10,000 person-years), and Japan (169 per 10,000 person-years).^7^^,^^9^^,^^10^ Conversely, the incidence of pneumonia in Mongolia was higher than that of other LMICs in Asia, including Vietnam (8·1 per 10,000 person-years) and Malaysia (15·9 per 10,000 population).^21^^,^^22^ Several factors may contribute to this difference. First, community-acquired pneumonia surveillance data are limited in many LMICs, and discordance between study case definitions and data sources can make it difficult to compare incidence rates.^2^ Second, Mongolia's age distribution differs from that of high-income countries. In Mongolia, <5% of the population is ≥65 years old. Comparatively, between 17-28% of the populations in the USA, Germany, and Japan are ≥65 years.^23^ In Malaysia and Vietnam, the proportions of those ≥65 years are higher than in Mongolia (∼7–8%), but still lower than most high-income countries.^23^ After age-standardizing the incidence of community-acquired pneumonia in Japan, incidence rates decreased by one-third in individuals aged 15 years or older.^10^ Therefore, it is plausible that Mongolia's comparatively low incidence of pneumonia can be partially attributable to varying age distributions between LMICs and higher-income countries. Third, our study targeted adults admitted to district hospitals and did not include adults who sought care at primary health care centers, were admitted to tertiary hospitals, or who never sought treatment. It is unlikely that a large proportion of pneumonia cases were missed from tertiary hospitals, as they are considered specialty facilities that typically require a referral from a secondary hospital. However, out-of-pocket expenses, self-medication, and dissatisfaction with healthcare services remain important barriers in deterring adults from seeking care.^24^^,^^25^ Our study found that patients who received antibiotics within 48 hours prior to hospital admission had lower odds of severe pneumonia, indicating that healthcare utilization practices and delay in seeking care may contribute to adverse health outcomes.^24^^,^^26^^,^^27^ While nearly all Mongolians have social health insurance and reform efforts have been made to provide equitable access healthcare, financial barriers persist.^25^^,^^26^ Despite typically having greater health needs, lower income individuals are more likely to use primary or public secondary care services, whereas higher income individuals are more likely to use secondary or tertiary services regardless of health needs and underestimating the true burden of community-acquired pneumonia.^25^^,^^27^

We found seasonal variations in clinical pneumonia hospitalizations, with the highest incidence rates occurring during the winter months. These results were expected, as pneumonia is considered highly seasonal in Mongolia, where winter temperatures can reach as low as −40°C. During the winter months, Ulaanbaatar's air pollution levels reach well beyond World Health Organization and national guidelines, potentially compounding the pneumonia burden in the population.^15^^,^^28^ In an effort to improve air quality in Ulaanbaatar, the Mongolian government banned the consumption of raw coal in 2019. While early reports indicate a notable reduction in particulate matter concentrations, more research is needed to quantify the impacts on the burden of community-acquired pneumonia and other diseases.^28^

The annual incidence for clinical pneumonia and severe pneumonia hospitalizations in adults >65 years was at least three times higher than other age groups, indicating the importance of age as a risk factor for pneumonia morbidity. Several biologically plausible factors may explain these findings. First, the natural ageing process leads to immunosenescence, making it more difficult to fight off infection over time.^3^^,^^29^ Second, comorbidities are important risk factors for developing community-acquired pneumonia, with older adults typically having more comorbidities than younger adults.^3^^,^^30^ Several underlying medical conditions, including COPD, tuberculosis, cirrhosis or liver failure, coronary artery disease, heart failure, chronic renal failure, and diabetes were independently associated with severe pneumonia in our study. While hypertension was the most common documented underlying medical condition in this study, it is not generally considered a risk factor for pneumonia and was not a risk factor for severe pneumonia.^31^^,^^32^

As LMICs progress and become increasingly urbanized, they develop dual burdens of disease, where non-communicable diseases (NCDs) rapidly increase while the infectious disease burden remains high.^33^^,^^34^ Over the last 30 years, Mongolia has undergone such demographic and epidemiological transitions.^33^^,^^35^ Strengthened health systems and improved environmental conditions have led to increases in average life expectancy and decreases in some communicable disease burdens.^26^^,^^35^ Meanwhile, the burden of chronic NCDs has notably increased.^35^ As a result, individuals with underlying medical conditions are at higher risk for infectious diseases, including community-acquired pneumonia.

Our study found that males were more likely to develop severe pneumonia. Gender differences in occupation, tobacco and alcohol use, and other environmental exposures likely contribute to the severity of community-acquired pneumonia in men.^6^^,^^14^ Men generally participate in more outdoor manual labor than women, increasing their exposure to occupational air pollutants and ambient air pollution.^15^^,^^36^ Exposure to silica and other industrial dusts increases the rates of pneumoconiosis, occupational respiratory diseases, and lung cancer in coal mine, power plant, construction, and manufacturing workers.^36^^,^^37^ Furthermore, almost half of the male population smokes tobacco and ∼50% drink alcohol, compared with <10% and 30% of the female population, respectively.^13^^,^^38^ Men are also less likely to use outpatient services and may delay seeking care for communicable diseases or other health conditions considered less urgent.^24^^,^^27^ Consequently, Mongolia has one of the largest life expectancy gaps between males and females globally, with women expected to outlive men by almost a decade.^35^ Risk factor data collected as part of the prospective surveillance study will provide additional insights into the burden of disease in men.

The case-fatality ratio in our study was low compared with other LMICs.^2^ Because this study was conducted through a retrospective chart review, we were only able to capture deaths reported during hospital stay and did not follow up with patients after hospital transfer or discharge, likely underestimating the true proportion of pneumonia-related deaths in Ulaanbaatar. Additionally, over half of all patients were discharged with sequelae, possibly resulting in a small number of undocumented deaths. Furthermore, higher income individuals typically overutilize inpatient services, whereas lower income individuals underutilize inpatient services due to financial constraints.^25^, ^26^, ^27^ As a result, different thresholds for hospitalization among different income groups could contribute to an underestimate of the case-fatality rate.

In many LMICs, including Mongolia, chest radiographs, blood cultures, and other diagnostic tests are usually not a part of routine care for adult pneumonia case management due to economic and logistical constraints. As a result, we relied on clinical assessment and did not require diagnostic test results for study inclusion. While this may be seen as a limitation, we do not believe this alters the validity or interpretation of our results for several reasons. First, our case definition was derived from the clinical assessment inclusion criteria in the Etiology of Pneumonia in the Community (EPIC) study, which found high congruence (93%) between a treating clinician's clinical assessment and radiographic evidence of pneumonia.^7^ Second, a systematic review found that while individual clinical signs or symptoms have a limited ability to be the sole predictor of community-acquired pneumonia, clinical decision tools that include multiple clinical features and biomarkers may be useful in diagnosing pneumonia.^39^ Third, while chest radiographs can be important tools in confirming a pneumonia diagnosis, they are considered to be less reliable for elderly patients and for those with dehydration.^40^^,^^41^ Additionally, at least two systematic reviews have found chest radiographs to be imperfect diagnostic tools that are not 100% sensitive when diagnosing pneumonia.^42^^,^^43^ Fourth, while blood cultures can be useful in determining pneumonia etiology, identifying a causal pathogen continues to be a challenge globally due to low rates of pathogen detection.^7^ Fifth, we were unable to test for urea nitrogen in the blood, which limited our ability to scale pneumonia severity using traditional criteria, including the pneumonia severity index and CURB-65. However, studies from LMICs have found mixed evidence to support the validity of these tools in estimating inpatient mortality in resource-limited settings.^2^

There are several limitations to our study in addition to those already discussed. First, because our data were collected through retrospective chart review, some data were missing from patient records and not all relevant risk factors could be assessed. While our sensitivity analysis estimated the upper limit of what the incidence may be if all community-acquired pneumonia admissions with inadequate medical records had met our case definition, the results demonstrated how missing data could bias the analyses. Results from the prospective surveillance data will provide additional insights into better understanding the determinants and etiology of community-acquired pneumonia in Mongolia. Second, incidence rates calculated for hospitalized patients may underestimate the total community-acquired pneumonia incidence in Ulaanbaatar. In cases of mild or moderate community-acquired pneumonia, adults might choose to seek care at their local family health clinic or as an outpatient at the hospital, or choose to not seek any care for their symptoms.^24^^,^^44^ Third, we were unable to track mortality after patients were discharged, as comprehensive vital registration data are currently limited in Mongolia.^35^ Future studies should consider including follow up periods post-discharge to community-acquired pneumonia surveillance programs to better understand the mortality rates in adults. Fourth, we noted increases in the annual pneumonia incidence over a short timeframe which was surprising. Though we cannot determine the reason for this increase, it is possible that improvements in healthcare utilization or in the healthcare system may have contributed to this trend. Finally, the results of this study are not generalizable to all adults in Mongolia, as it was conducted in the capital city of Ulaanbaatar and did not cover mining districts. Ulaanbaatar is the most densely populated city in Mongolia. Subsequently, the city's rates of respiratory diseases may not be translatable to all other provinces (aimags) in Mongolia.^14^^,^^28^

To our knowledge, this is the first systematic surveillance program examining community-acquired pneumonia in adults in Mongolia. Future studies should examine community-acquired pneumonia etiology and the extent to which important risk factors, such as air pollution and socioeconomic status, impact adult pneumonia morbidity and mortality. Early intervention methods, such as introducing pneumococcal disease and influenza immunization programs for older adults and those with underlying medical conditions, should also be considered. In the coming years, Mongolia's elderly population is expected to more than double, likely resulting in increased rates of chronic NCDs and intensifying demands on the country's healthcare system.^27^ By establishing the adult burden and clinical characteristics of community-acquired pneumonia, targeted interventions focusing on high-risk groups can be developed to have a more meaningful impact on the burden of disease.

## Contributors

Conception/design of the work: EKM, CVM; Responsible for study oversight: CVM, TM, BDG, EMD, BT; acquisition of data: MU, BS, DL, DN, BP, TM; analysis of data: KF; drafting of the manuscript: KF. All authors were involved in data interpretation and review of the final manuscript.

## Data sharing statement

All relevant data are within the paper and the supplementary information files.

## Declaration of interests

EKM and CVM are lead investigators of this study through Murdoch Childrens Research Institute, which was funded by Pfizer. MU, CDN, BS, DL, BP and TM are investigators on this collaborative research project funded by Pfizer. BDG and EMD are employees of Pfizer and own Pfizer stock or stock options. The other authors have no relevant conflicts of interest to declare. This study is a Murdoch Childrens Research Institute-sponsored study which is investigator-led, and funded under a collaborative agreement by Pfizer Inc.

## Appendix Supplementary materials

Image, application 1
