# Supplementary material for: Epidemiology of pneumonia in hospitalized adults ≥18 years old in four districts of Ulaanbaatar, Mongolia, 2015–2019
Source: Lancet Reg Health West Pac. 2022 Sep 10;30:100591. doi: 10.1016/j.lanwpc.2022.100591 (PMC9677069; doi:10.1016/j.lanwpc.2022.100591)
Supplement: Supplementary file 1 [file mmc1.pdf]

Supplementary appendix

Epidemiology of pneumonia in hospitalized adults ≥18 years old in four districts of Ulaanbaatar, Mongolia, 2015—2019

Supplementary Table 1. Sensitivity analysis for incidence rates for clinical pneumonia hospitalizations, include patients with a pneumonia diagnosis at admission but inadequate medical records for study inclusion, by age in adult ≥18 years of age in Ulaanbaatar, Mongolia, January 2015—December 2018 (per 10,000 population)

	**2015**<br>**IR (95% CI)**	**2016**<br>**IR (95% CI)**	**2017**<br>**IR (95% CI)**	**2018**<br>**IR (95% CI)**
18-25 years	11.1 (9.3-13.1)	11.4 (9.5-13.5)	9.8 (8.0-11.8)	14.4 (12.1-16.9)
26-45 years	12.6 (11.3-13.9)	11.1 (10.0-12.3)	11.1 (10.0-12.2)	13.8 (12.6-15.1)
46-65 years	19.4 (17.2-21.8)	27.9 (25.3-30.8)	27.1 (24.6-29.8)	25.2 (22.9-27.8)
>65 years	63.0 (54.4-72.7)	85.5 (75.7-96.3)	81.1 (71.7-91.4)	80.2 (71.0-90.2)
All ≥18 years	*16.4 (15.4-17.4)*	*19.0 (17.9-20.1)*	*18.4 (17.4-19.5)*	*20.5 (19.4-21.6)*

IR= Incidence rate; CI = Confidence Interval
